# Influence of Social Distance Expressed by Driving Support Agent’s Utterance on Psychological Acceptability

**DOI:** 10.3389/fpsyg.2021.526942

**Published:** 2021-02-24

**Authors:** Tomoki Miyamoto, Daisuke Katagami, Yuka Shigemitsu, Mayumi Usami, Takahiro Tanaka, Hitoshi Kanamori, Yuki Yoshihara, Kazuhiro Fujikake

**Affiliations:** ^1^Graduate School of Tokyo Polytechnic University, Kanagawa, Japan; ^2^Faculty of Engineering, Tokyo Polytechnic University, Kanagawa, Japan; ^3^National Institute for Japanese Language and Linguistics, Tokyo, Japan; ^4^Institutes of Innovation for Future Society, Nagoya University, Aichi, Japan; ^5^School of Psychology, Chukyo University, Aichi, Japan

**Keywords:** human-agent interaction, human-robot interaction, driving support agent, politeness theory, social distance, utterance design, psychological acceptability

## Abstract

In this study, we discuss the psychological acceptability of an utterance strategy used by the *D*riving *S*upport *A*gent (DSA). Previous literature regarding DSA suggests that the adoption of a small robot as a form will increase acceptability. However, the agent’s utterance has been reported as a problem faced by the user. Therefore, in this study, we designed the agent’s utterance using politeness strategy as described by Brown and Levinson’s famous sociolinguistics and pragmatics theory and analyzed its acceptability through a participant-based experiment. In this experiment, we used positive and negative politeness strategies (PPS and NPS, respectively). In general, PPS is utilized to reflect the desire to be liked/recognized by others, whereas NPS is utilized to reflect the need for not wanting to be disturbed by others. Based on our results, PPS was rated high compared to NPS (*n* = 197). Therefore, many participants highly evaluated PPS. However, there was a group of participants who appreciated NPS. There were also participants who evaluated the two strategies equally. The number of participants in these three groups was observed at 4:1:1. This result contributes as an index on the utterance design of the DSA.

## Introduction

Cars are an important method of transportation for many people. However, life-threatening road accidents often occur due to misjudgment/misoperation by the driver. A report published by the World Health Organization (2018) shows that approximately 1.35 million people worldwide have been killed annually due to automobile-related road accidents. Therefore, various researchers are making efforts to reduce the rate of road accidents through various technological inventions. A representative example is the development of automatically driven cars (i.e., level five) in which the system handles all of the driving operations. If such cars become widespread, it might help to eliminate road accidents due to human errors. However, a country’s laws and ethics need to be changed to include fully automatically driven cars, for example, the degree of acceptance of automatically driven cars by the people and taking responsibility in an event of an accident (Hulse et al., 2018; Nunes et al., 2018; Polidori et al., 2018; Meder et al., 2019; Taeihagh and Lim, 2019). Therefore, although the technological progress in the field of automobile engineering is remarkable, it might take more time to popularize fully automatically driven cars among people.

In recent years, with the goal of enabling a new kind of relationship between humans and cars, there is an increasing amount of research on *D*riving *S*upport *A*gent (DSA) (e.g., robot/virtual character) loaded in a car, which acts like a user’s partner (Tanaka et al., 2018a, b; Karatas et al., 2019; Lee et al., 2019; Miyamoto et al., 2019). These agents have intelligent utterance functions and support users by voice utterances, hand gestures, and facial gestures. In general, the DSA is intended to assist the user’s recognition/judgment (e.g., assist the driver in driving safely by understanding the user’s preferences, favorite places, fuel requirement, traffic issues, and so on). Thus, we can say that the DSA is a support aimed at a user who performs his driving operation himself, which is considerably different from an automatically driven car. According to a previous study, small robots that speak a synthesized speech are more acceptable (i.e., not bothersome, not hinder for driving) than that of virtual characters and synthesized speech-only agent (Tanaka et al., 2018a). This information was obtained via a survey questionnaire and gaze behavior analysis. Also, small robots in this context provide assistance only by voice utterances and hand gestures (e.g., pointing left and right). It does not make facial gestures and does not move around on the dashboard. This is because it is dangerous if these actions guide the driver’s line of sight. Therefore, in this study, we consider that a small robot that speaks speech is suitable as a form of DSA. In addition, the demand for a DSA in Japan is particularly high. Therefore, in Japan, research and development is in progress with regard to the practical application of DSA (Tanaka et al., 2019).

A DSA that performs voice utterances is considered to be classified as a task-oriented agent. However, the knowledge regarding the utterance design of a task-oriented agent, such as DSA, Siri, and Cortana–in which the user can accept the utterance–has not been established. A study by Luger and Sellen (2016) investigated the previously unknown effects of Siri, Google Now, and Cortana’s utterance strategies on the psychology of users. The investigation revealed that there was a problem related to the task-oriented agent’s utterance. For example, the user rejects the agent if the agent’s utterance content falls far short of the user’s expectations (Luger and Sellen, 2016). Therefore, it is important to examine the utterance design of task-oriented agents that support users in their daily lives. Furthermore, studies on DSAs have shown that DSAs improve their receptivity by supporting the user through voice utterances, but there are certain shortcomings. Specifically, Japanese research has shown that users feel annoyed and disgusted with the utterances of DSAs (Fujikake et al., 2017). This problem occurred because Fujikake et al. (2017) had not examined the effect of DSA utterance content on users’ psychology (Fujikake et al., 2017). During the DSA–user interaction, if a user feels frustrated due to the behavior of the DSA, then it might affect their driving negatively. Therefore, utterance strategies that enhance the acceptability of DSAs need to be designed.

In studies related to human–agent and human–robot interactions, and topics that deal with artificial media that conduct social interaction with humans, politeness theory (Brown and Levinson, 1987) is seen as an approach to design utterance that is easily accepted by users (Salem et al., 2013; Torrey et al., 2013; Srinivasan and Takayama, 2016; Miyamoto et al., 2017, 2019; Lee et al., 2019). Politeness theory is a well-known framework on conversation in the fields of pragmatics and sociolinguistics. For example, Miyamoto et al. (2017) applied an utterance strategy that increased the parties’ closeness, which facilitated the construction of a smooth relationship with the other party. In particular, agent utterances were designed based on negative politeness and positive politeness strategies (NPS and PPS, respectively). The effects of PPS and NPS were compared by participant experiments. Herein, NPS is an utterance strategy that maintains closeness with a conversation partner by apologizing, using fuzzy opinion, and so on to reflect the partner’s need for not wanting to be disturbed, whereas PPS is an utterance strategy that increases closeness with a conversation partner by compliments, includes a partner in action, and so on to reflect the partner’s desire to be liked/recognized (Brown and Levinson, 1987). In Miyamoto et al.’s study (2017), PPS is an utterance strategy that increases closeness with another party through joking behavior. Miyamoto et al. (2017) assume non-task-oriented conversation scenes for Japanese between human and agent. However, the general DSA behaves for a task as driving a car. In English and Korean language studies, the efforts to use PPS and NPS for task-oriented utterance design such as robots are attracting greater attention (Salem et al., 2013; Torrey et al., 2013; Srinivasan and Takayama, 2016; Lee et al., 2019).

Lee et al. (2019) designed a DSA’s utterance using politeness and verified the effect by conducting an experiment with a participant. According to their results, the implementation of polite utterance using NPS was found to be satisfactory. Therefore, their result suggests that it is important to consider social factors in an utterance design of a DSA. However, in their study, 26 out of 56 participants did not have a driver’s license. Therefore, 46.4% of the participants in Lee et al.’s study have never driven a car. In their study, the agent does not have a physical form. It has been reported that people are significantly more receptive of small robots than agents without physicality (Tanaka et al., 2018a). On the other hand, Miyamoto et al. (2019) conducted an utterance design of a small robot as the DSA based on the politeness strategy; however, their participants were limited to students. The video used in the experiment as stimulus presentation to experimental participants observes the interaction between a user and an agent from a third-party perspective, which means it has not been evaluated from the driver’s perspective. These previous studies provided useful knowledge into the design of DSA utterances, but the experiment has its drawbacks. Additionally, Lee et al. (2019) and Miyamoto et al. (2019) reported different findings. Lee et al. (2019) showed that NPS was accepted by users, while Miyamoto et al. (2019) reported that PPS was more acceptable to users than NPS. Furthermore, neither study verified the validity of DSA utterances in terms of politeness theory. Therefore, the acceptability of NPS/PPS in the design of DSA utterance is questionable.

In this study, we aimed to examine the relationship between the psychological acceptability of a DSA and the social factors that are expressed by an agent’s utterance (i.e., PPS vs. NPS). In our experiments, we resolve certain issues that had not been adequately addressed in previous studies (Lee et al., 2019; Miyamoto et al., 2019). Specifically, the emphasis will be on obtaining data on the acceptance of DSA by experimental participants with a driver’s license. So, the video was created as a stimulus presentation for the experimental participants listening the agent utterances from the driver’s perspective. The reason for using videos for experiments is to get as much experimental data as possible. The validity of the utterance is confirmed by a discussion between politeness theory specialist researchers. This study implements a DSA utterance that expresses social factors using a politeness strategy. We focus on social distance from among the social factors in this study. Specifically, PPS was implemented as a situation in which the DSA estimated that the social distance to the user was short, and NPS was implemented as a situation in which the DSA estimated that the social distance to the user was long. Then, we compared the acceptability of DSA utterances. This gives us knowledge that contributes to the utterance design of agents acting as user partners. The new findings that this study provides for the design of DSAs are as follows. We believe that these findings are more robust than previous studies in terms of the number/quality of participants and the validity of the politeness utterances in the experiment.

• The ratio of users who prefer PPS, users who prefer NPS, and users who evaluate the two strategies equally is 4:1:1.
• PPS significantly increases the anthropomorphism and animacy of the DSA compared to NPS.
• There is a strong positive correlation between the user’s perceived intelligence of the DSA and driving support acceptance evaluations of the DSA.

Also, in this study, we used Japanese language in the utterance design of the agent because of the growing need for DSA in Japan (Tanaka et al., 2019). However, future studies can be targeted with users with different cultural backgrounds and with other languages.

This study is presented as follows. Chapter 2 gives an overview of the politeness strategy (Brown and Levinson, 1987) and its application in this study. In Chapter 3, we explain how to design DSA’s utterances and how to create videos for the evaluation. In Chapter 4, an experiment is performed using the created video. In Chapter 5, we show the experimental results. Chapter 6 discusses the experimental results, and finally, Chapter 7 presents the conclusion.

## Politeness Theory

### Brown and Levinson’s Politeness Strategies

Of the two individuals interacting with one another, we define the speaker as *S* and the listener as *H*, based on the work of Brown and Levinson (1987). According to Brown and Levinson (1987), both *S* and *H* desire to form an interpersonal relationship with one another. This desire is called “face” (Goffman, 1967) and is classified as either a negative face or a positive face. A negative face is the desire to separate and be independent from others, whereas a positive face is the desire to be favored by others. In general, *S* wishes to preserve *H*’s face during dialogue. However, depending on the action, the result may threaten *H*’s face. Such an action is called a face-threatening act (FTA). When *S* needs to perform an FTA toward *H*, *S* estimates the weight of the FTA. Here, the weight of the FTA is calculated as per the following equation (Brown and Levinson, 1987).

(1) Wx=D(S,H)+P(H,S)+Rx

In Eq. 1, *D* is a value that indicates the social distance between *S* and *H*, *P* is the amount of force *H* exerts on *S*, and *Rx* is a value that indicates how burdensome the FTA is perceived to be within the two parties’ specific cultural context. More specifically, the weight (*Wx*) of the FTA is the sum of *D*, *P*, and *Rx*. Since *P* and *Rx* fluctuate across cultures, the weight of the FTA also varies depending on the given culture, even if utterance is identical. *S* chooses a politeness strategy according to *Wx*. The most representative politeness strategies are PPS and NPS. PPS is selected by *S* when *Wx* is relatively low (i.e., *H* has a positive face). Conversely, if *Wx* is high (i.e., *H* has a negative face), *S* chooses NPS. Table 1 shows all 10 strategies for NPS and 15 strategies for PPS. *S* uses these strategies in conversation to build good relationships with the *H*.

**TABLE 1:** Politeness strategies (Brown and Levinson, 1987).

PPS	NPS
1: Notice, attend to *H* (his interests, wants, needs, goods)	1: Be conventionally indirect
2: Exaggerate (interest, approval, sympathy with *H*)	2: Question, hedge
3: Intensify interest to *H*	3: Be pessimistic
4: Use in-group identity markers	4: Minimize the imposition, *Rx*
5: Seek agreement	5: Give deference
6: Avoid disagreement	6: Apologize
7: Presuppose/raise/assert common ground	7: Impersonalize *S* and *H*: Avoid the pronouns “I” and “you”
8: Joke	8: State the FTA as a general rule
9: Assert or presuppose *S*’s knowledge of and concern for *H*’s wants	9: Nominalize
10: Offer, promise	10: Go on record as incurring a debt, or as not indebting *H*
11: Be optimistic	
12: Include both *S* and *H* in the activity	
13: Give (or ask for) reason	
14: Assume or assert reciprocity	
15: Give gifts to *H* (goods, sympathy, understanding cooperation)	
	

### Interpretation of Politeness Strategies

In this study, we focus on *D* from among the social factors. The politeness strategies also affect social factors other than *D* (i.e., *P* and *Rx*). However, *P* is fixed in the interaction between the user and the DSA in this study. Specifically, since the DSA is a tool that supports users, it has a smaller *P* than users. Also, *Rx* does not change in this study because it restricted to the Japanese context. Therefore, in this study, *P* and *Rx* are fixed in the relationship between users and the DSA, and the DSA changes representation of *D* (estimate) through PPS/NPS.

According to the section “Brown and Levinson’s Politeness Strategies,” the politeness strategy (Brown and Levinson, 1987) plays an important role in the smooth communication between two parties. According to Usami (2002) and Kiyama et al. (2012), politeness theory can also be applied to non-Western cultures. However, the primary subject of politeness theory is the language of Western culture. Therefore, we will discuss how to handle PPS/NPS according to a target language. By considering how to handle politeness strategies according to the language used by agents, discussions can be made according to culture.

**FIGURE 1:**
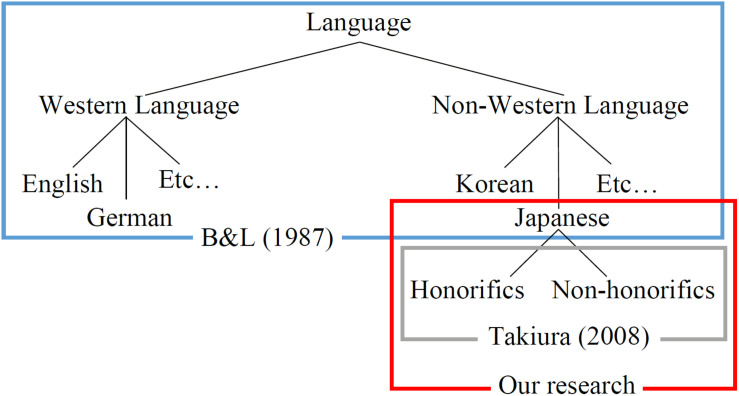
Classification of politeness research.

Figure 1 shows the classification of politeness research in Western language/non-Western language. The politeness theory (Brown and Levinson, 1987) also covers non-Western language, but an effect of Japanese honorifics on a face is not described in detail. According to Takiura (2008), honorifics in Japanese generally represents a remoteness of *D*, similar to NPS, and non-honorifics generally represent the closeness of *D*, similar to PPS. Therefore, honorifics/non-honorifics is important to express *D* by using Japanese. Therefore, in this study, in addition to PPS/NPS defined by Brown and Levinson (1987), Japanese sentence ending expressions (honorifics/non-honorifics) are included in the politeness strategy. Furthermore, in this study, in order to clarify the difference in *D* expressed by PPS and NPS, end of sentence of the agent’s utterance that used NPS is designed honorifics, and end of sentence of the agent’s utterance that used PPS is designed non-honorifics.

Based on Brown and Levinson (1987), we discuss the effect of PPS and NPS on the *D* between *S* and *H*. Here, the closeness (*C*) between *S* and H is defined as follows.

(2) C=-D

In other words, the smaller the *D* between *S* and *H*, the higher the intimacy between *S* and *H*. *C* is expected to change over time. For example, from the time when *S* and *H* first meet (*t* = 0), *S* influences *H*’s face through politeness strategy (PPS/NPS), and *C* changes immediately after that (*t* = 1). However, here, following Brown and Levinson (1987), the change in *C* in one utterance unit is the subject of discussion. In other words, this paper does not consider the integral value of *C* in a long-term conversation. Based on the above, we define the closeness (*C*_*t*_) between *S* and *H* at a certain time (*t*) as follows.

(3) $$C{}_{t}=F{}_{t-}{}_{1}-T{}_{u},{}_{t-}{}_{1}$$

In Eq. 3, *F* is the degree to which *H*’s face is satisfied, and *T*_*u*_ is the degree to which *H*’s face is threatened by *S*’s utterance (*u*: *PPS*, *NPS*). According to Brown and Levinson (1987), *H*’s face is threatened to some extent by PPS/NPS by *S*, so *T*_*u*_ is positive (*T_*u*_* > 0). If *S* chooses a politeness strategy (PPS or NPS) with a small *T_*u, t*__–__1_*, *C*_*t*_ will be relatively high since the violation of *H*’s face can be minimized. In this case, if *C*_*t*_ ≥ 0, then *H* is considered to be in a comfortable state at *t*. In other words, the relationship between the value of *C*_*t*_ and the state of *H* is as follows.

• *C*_*t*_ ≥ 0 ↔ *H* feels Comfort at *t* (↔: Necessary and sufficient conditions)
• *C*_*t*_ < 0 ↔ *H* feels Discomfort at *t*

The type of face that *H* has (positive face/negative face) varies according to the relationship between the value of *C*_*t*_ and the threshold of the face (*θ_*F*_*) (Brown and Levinson, 1987). In this case, the value of *T_*u*__, t_* varies as follows.

• *C*_*t*_ > *θ_*F*_* (i.e., *H* has Positive Face) ↔ *T_*PPS*__, t_* < *T_*NPS*__, t_*
• *C*_*t*_ < *θ_*F*_* (i.e., *H* has Negative Face) ↔ *T_*PPS*__, t_* > *T_*NPS*__, t_*

In other words, the effect of PPS/NPS changes depending on the type of face that *H* has. In this paper, in order to investigate the psychological effects of PPS and NPS by DSA on users, we set up a condition in which *C* between DSA and users is estimated to be high (PPS condition) by DSA and a condition in which *C* is estimated to be low (NPS condition) by DSA in a within-subjects design.

## Materials and Methods

In this study, the utterance of the DSA is designed based on PPS/NPS, and the psychological acceptability is verified. In particular, emphasis is placed on obtaining evaluation data from many participants; therefore, we used videos to evaluate. In the following, utterances and videos of DSA used for evaluation are described.

### Driving Situations

**FIGURE 2:**
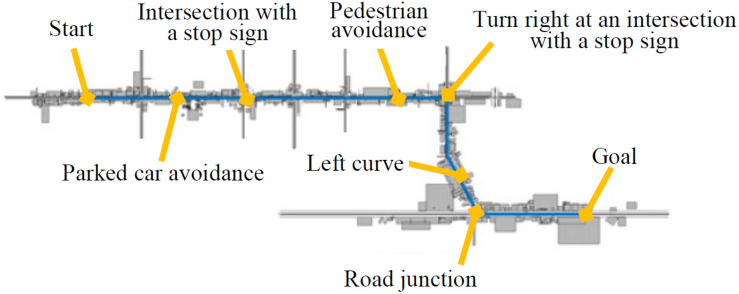
Experimental course (Tanaka et al., 2018a).

There are various situations that a driver encounters while driving a car. However, it is considered that evaluating an acceptability of the agent’s utterances for all the possible driving situations would increase the cost of experiments. We also consider that agents should not speak in situations where the driver is driving at a very high cost. Even in the preliminary survey, in the actual vehicle environment, the situation and frequency of utterances by the driving support agent are strongly restricted (Tanaka et al., 2020). The most important task for a driving support agent is to encourage the driver to drive safely, but in a high-cost situation where it is difficult to accept comments from others even if the agent makes full use of the politeness strategy, there is a risk of its adverse effects on driving. Therefore, in this study, the driving situations to be evaluated in the experiment are limited in order to reduce the cost (restraint time, fatigue, etc.) of the experimental participants. Specifically, in this experiment, it is assumed that experimental participants drive the experimental course used in Tanaka et al. (2018a). Figure 2 shows the simulated driving course used by Tanaka et al. (2018a). This course is a reproduction of the road around Nagoya University in Japan. In this study, we designed and evaluated agent utterances for a parked car avoidance, intersection with a stop sign (go straight/turn right/left), a pedestrian avoidance, and a left curve. In each driving situation, the agent speaks once.

### Utterance Strategies to Be Evaluated

**FIGURE 3:**
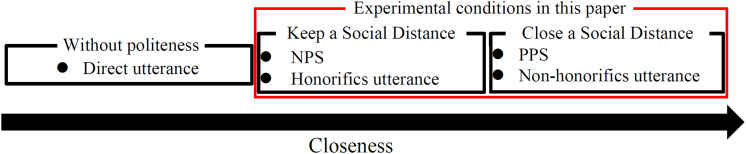
The relationship between politeness strategy and closeness in this study.

As described in the section “Politeness Theory,” politeness strategies are effective in manipulating *D* in interpersonal relationships, and *D* expressed by speech affects closeness (Brown and Levinson, 1987). Figure 3 illustrates the relationship between politeness strategies and closeness in this study. It indicates that the closer the *D*, the lower is the degree of face infringement assumed by *S* and the higher is the closeness expressed by *S*’s utterance. PPS shrinks the *D* between *S* and *H* if it is used when *H* has a positive face, and NPS maintains the *D* between *S* and *H* by minimizing the violation of *H*’s face if it is used when *H* has a negative face (Brown and Levinson, 1987). Therefore, PPS has a higher degree of closeness with *H* assumed by *S* and a higher degree of closeness when the utterance is accepted by *H* than NPS. Also, there are direct utterances that do not use politeness strategy (e.g., “*Slow down!*”). However, direct utterances have been reported to be less acceptable in driving support (Fujikake et al., 2017; Tanaka et al., 2018a) and significantly less acceptable than PPS/NPS (Salem et al., 2013; Torrey et al., 2013; Srinivasan and Takayama, 2016; Deshmukh et al., 2018; Lee et al., 2019). Previous studies have shown that direct speech is less receptive than PPS/NPS. Therefore, in this paper, direct utterance is not adopted as an experimental condition, and NPS condition and PPS condition are set as experimental conditions.

### Utterance Design

Herein, we designed an utterance of a DSA. First, as described in the section “Driving Situations,” an agent speaks for a parked car avoidance, intersection with a stop sign (go straight/turn right/left), a pedestrian avoidance, and a left curve. Of these, at an intersection with a stop sign, go straight/turn right/left are regarded as one category (i.e., driving situation). Next, utterances are created for strategies that are considered to be applicable to the driving situation that occurs in the course presented in Figure 2. The support provided by the agent is suggestions and instructions for correcting a user’s driving behavior. This was determined with reference to Tanaka et al. (2018a). For example, on the left curve in Figure 2, a deceleration instruction is given to encourage safe driving. Table 2 shows the PPS targeted in this study. All 10 NPSs were covered in this study. In addition, eight PPS strategies were considered: “Exaggerate (interest, approval, sympathy with *H*),” “Seek agreement,” “Presuppose/raise/assert common ground,” “Assert or presuppose *S*’s knowledge of and concern for *H*’s wants,” “Offer, promise,” “Be optimistic,” “Include both *S* and *H* in the activity,” and “Give (or ask for) reason.” We excluded the other seven strategies for the following reasons: Mainly used in non-task-oriented dialogue (i.e., “Notice, attend to *H* (his interests, wants, needs, goods),” “Intensify interest to *H*,” “Joke,” and “Use in-group identity markers”); for offering support itself (i.e., “Assume or assert reciprocity” and “Give gifts to *H* (goods, sympathy, understanding cooperation)”); and for responding to the other’s utterance (i.e., “Avoid disagreement”). Based on this, by using all NPS strategies and eight PPS strategies, a total of 72 utterances were created for four types of driving situations: a parked car avoidance, intersection with a stop sign, a pedestrian avoidance, and a left curve.

**TABLE 2:** In the case of PPS, the strategies evaluated by politeness theory specialist researchers and the strategies pre-excluded by the authors in the section “Utterances Design” were considered.

	Strategies	Reason of pre-excluded
Strategies evaluated by experts	2: Exaggerate (interest, approval, sympathy with *H*)	
	5: Seek agreement	
	7: Presuppose/raise/assert common ground	
	9: Assert or presuppose *S*’s knowledge of and concern for *H*’s wants	
	10: Offer, promise	
	11: Be optimistic	
	12: Include both *S* and *H* in the activity	
	13: Give (or ask for) reason	

Strategies pre-excluded by the authors	1: Notice, attend to *H* (his interests, wants, needs, goods)	Used in non-task-oriented conversations.
	3: Intensify interest to *H*	
	4: Use in-group identity markers	
	8: Joke	

	14: Assume or assert reciprocity	Used to offer support etc. itself.
	15: Give gifts to *H* (goods, sympathy, understanding cooperation)	

	6: Avoid disagreement	Used to reply to the other person’s utterance.

*The NPS had experts evaluate all strategies. As a result of the evaluation by experts, 2 strategies of PPS and 3 strategies of NPS were adopted as experimental stimuli.*

As described in the section “Interpretation of Politeness Strategies,” we designed the utterances based on Takiura (2008). We placed emphasis on the distant aspect of *D* by using NPS utterances as honorifics. In addition, the utterances using PPS were made non-honorific and emphasized a close aspect of *D*. However, we need to examine the validity of politeness strategies included in utterances. In this regard, we request two Japanese researchers specializing in politeness theory to evaluate the validity of the utterances. Both experts will evaluate the 72 utterances we created in three stages as follows: “no problem,” “substantially no problem,” and “problem” from the perspective of “whether it is correct as a politeness strategy.” The utterances were modified as necessary by politeness researchers. Based on the results of evaluation and discussions with politeness researchers, PPS and NPS were adopted for each five utterances so that the number of strategies was as many as possible. These utterances were selected from the 15 utterances that were rated “no problem” or “substantially no problem” by both politeness researchers. Five utterances were excluded to avoid overlapping politeness strategies as much as possible in the evaluation. It is important to suppress the influence on psychological acceptability caused by factors other than *D*. Specifically, three strategies of NPS (i.e., “Question, hedge,” “Minimize the imposition, *Rx*,” and “Apologize”) and two strategies of PPS [i.e., “Include both *S* and *H* in the activity” and “Give (or ask for) reason”] were adopted.

### Creating the Experimental Videos

**FIGURE 4:**
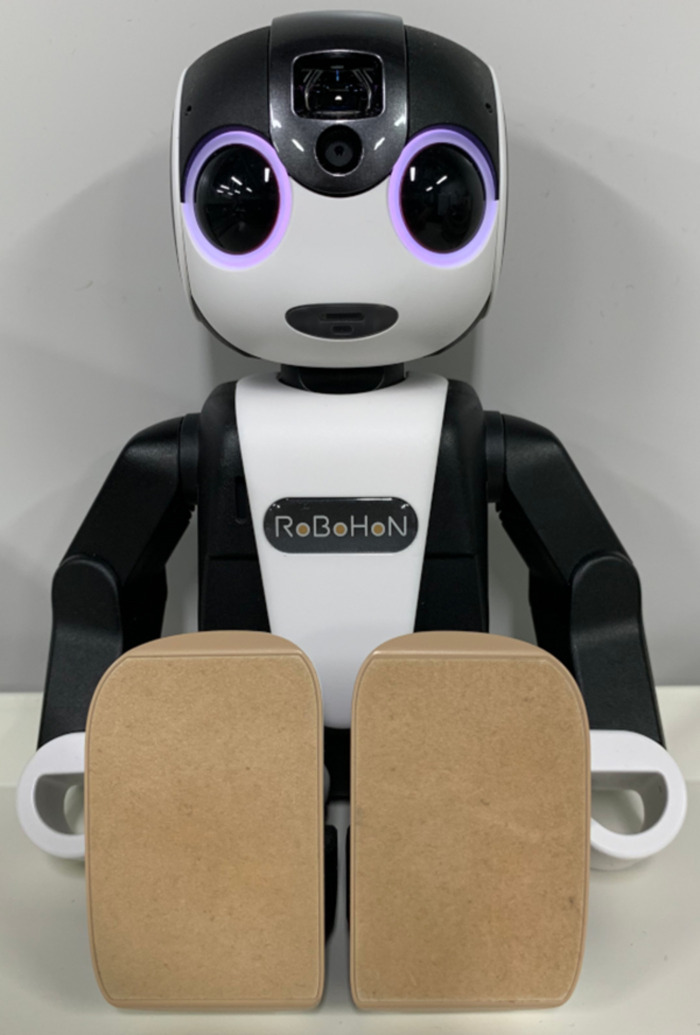
RoBoHoN (SHARP).

In this study, we created a video from a user’s perspective so that participants can feel as real as possible. The driving scene uses the video recorded in Tanaka et al. (2018a). This video was recorded by the drive recorder when the driving school’s instructor was driving the same course as presented in Figure 2. The video of the drive recorder was provided to us by Tanaka et al. We created a video of the DSA speaking and composited it with the video of the drive recorder. Aviutl^1^, a video editing software, was used for this work. We referred to Tanaka et al. (2018b) for the installation position and direction of the agent. RoBoHoN (SHARP) was used as the agent. Figure 4 shows the appearance of RoBoHoN, which is a small robot with a height of about 19.5 cm that can speak with the synthesized speech and has been used as a DSA by previous research (Tanaka et al., 2018b; Miyamoto et al., 2019). The voice of the RoBoHoN was constructed as per the following parameters: “5-year-old boy, innocent, cheerful, and diligent character” (SHARP).

**FIGURE 5:**
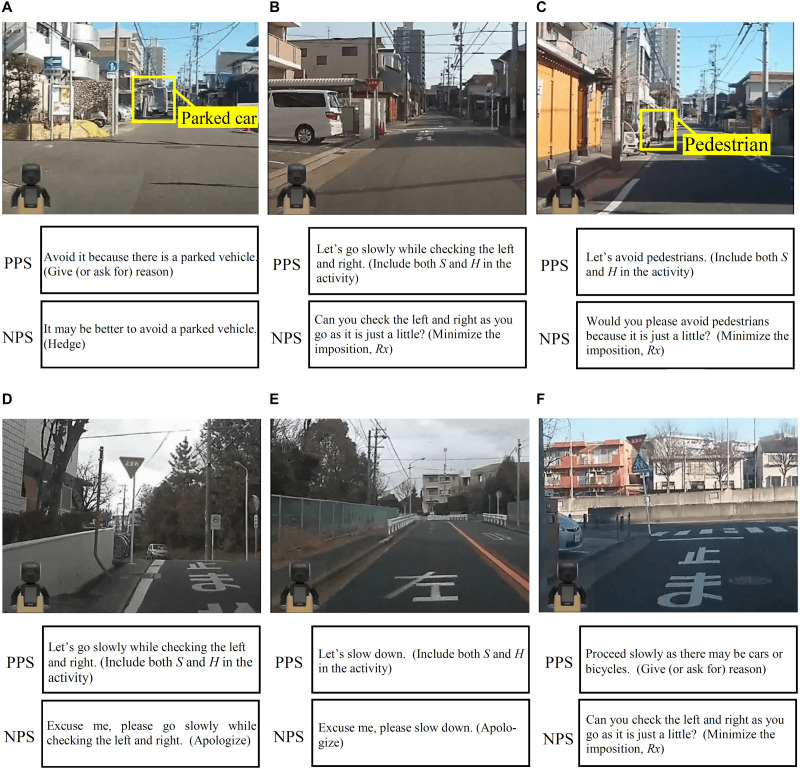
Experimental videos and utterances (strategy). **(A)** is “Parked car avoidance;” **(B)** is “Intersection with a stop sign;” **(C)** is “Pedestrian avoidance;” **(D)** is “Turn right at an intersection with a stop sign;” **(E)** is “Left curve;” and **(F)** is “Road junction.”

We created a video that the agent speaks using NPS and a video that utters using PPS. The agent’s utterance assumes that a user is driving the course, as shown in Figure 2. Figure 5 shows the created video image. Figures 5A–F correspond to the situation that occurs in the driving course shown in Figure 2 and play in this order. The utterance contents shown in Figure 5 are accompanied by the name of a politeness strategy used. The playback time of each video is about 2 min. The only difference between the two videos is the utterance content of the agent. The videos are shown as a Supplementary Video 1.

## Experiment

The purpose of this experiment is to verify the influence of social distance expressed by DSA’s utterance on psychological acceptability (i.e., PPS vs. NPS). To collect as many samples as possible, participants were recruited by crowdsourcing and an experiment was conducted in which participants watched the videos we created in the section “Utterances Design” (i.e., within-subjects design). The participants viewed the videos and answered the questionnaires on Google Forms^2^. The order of the videos to be viewed was counterbalanced by considering the order effect. The experiment was conducted based on the Research Ethics Guidelines for Humans of the Society of Automotive Engineers of Japan^3^.

### Procedure

First, a briefing is performed by presenting a text about the flow of the experiment to the experimental participants. Next, the text “This robot will support your driving by voice” is presented along with RoBoHoN images as an explanation of the robot used in this experiment. Furthermore, Figure 5A (without utterance text) was presented to the participants as an explanation of the position and orientation of RoBoHoN in the videos. In addition, we presented the following text to the participants: “The robot is in the car and sits in a place near the lower left of your front. Also, the robot sits facing forward as shown in the image below to check the surrounding situation. The same is true for the next video.” and “Please watch the video as if you were driving.” After that, the participants watch the videos that the agent speaks by using only NPS and using only PPS. Labels (A,B) are attached to the two videos. Participants in the experiment watched videos A and B in that order. To offset the order effect of the PPS condition and the NPS condition, the experimental participants were randomly divided into a group with the PPS condition as video A and a group with the NPS condition as video A. At the end of the experiment, the participants answered the agent evaluation questionnaire. At this time, the participants were instructed to compare and evaluate the impressions of the agents in videos A and B. Also, the participants in the experiment were instructed to answer the questionnaire in an intuitive manner. As the last question of the questionnaire, the participants will answer the following questions with two choices: Yes/No “The agent’s wording were different at the two videos. Did you notice about it?” The experiment ends when the participants answer all the questions in the questionnaire.

### Evaluation Items

#### Acceptability Evaluation of Driving Support

In this experiment, we used the questionnaire by Tanaka et al. (2018b) and adopted the seven-point Likert response scale system (1: *Perfectly not agree*; 2: *Hardly agree*; 3: *Pretty much not agree*; 4: *Neither*; 5: *Pretty much agree*; 6: *Almost agree*; and 7: *Perfectly agree*) to obtain responses. The following nine items were evaluated:

Q1: Favorability
Q2: Reliability
Q3: Familiarity
Q4: Want to use
Q5: Usability
Q6: Contribution for safe driving
#Q7: Uncomfortable
#Q8: Annoyance
#Q9: Disturbance

Of these, Q7, Q8, and Q9 with “#” are inverse items. For example, in Q7, as the evaluation value approaches seven, the numbers are reversed so that the evaluation is “Not uncomfortable.”

#### Impressions Based on General Evaluation Items for Social Robots

The Godspeed questionnaire developed by Bartneck et al. (2009) can investigate general anthropomorphism, animacy, likeability, perceived intelligence, and perceived safety as evaluation items for robot. In this study, we focused on anthropomorphism, animacy, and perceived intelligence. We consider that these are difficult to evaluate on the questionnaire shown in the section “Acceptability Evaluation of Driving Support.” The participants respond on the five-point Semantic Differential method for items that evaluate each factor (Bartneck et al., 2009).

### Participants

The 222 users of Crowd Works^4^, a famous crowdsourcing service in Japan, participated in this experiment. One problem in experiments using crowdsourcing is the possibility that the crowdsourcer may do a lax job (Burmania et al., 2015; Jonell et al., 2020). In this experiment, we used the results of responses to the question “The agent’s wording was different at the two videos. Did you notice about it?” which was used as an indicator to check whether the participants watched the videos. In each experimental condition, the sentence ending styles are different, which is perceived as a clear difference by Japanese speakers. Therefore, we assumed that the participants who did not notice the difference in wording did not watch the videos carefully. Thus, a total of 24 participants were excluded who responded that they did not notice the difference in the wording of the agent from the participants. Also, one participant whose responses were incomplete was excluded. In other words, 197 people [male: 103, female: 94, average age = 38.2 years, standard deviation (*SD*) = 9.6] are the subjects of analysis in this experiment. The participants in this experiment had a valid car driving license. After the experiment, participants received an incentive (i.e., 300 yen).

## Results

### Manipulation Check

Based on the score of “Unfriendly-Friendly” items in the Godspeed questionnaire (i.e., one is unfriendly; five is friendly), PPS [Mean (*M*) = 3.5, standard error (*SE*) = 0.07] gave a friendlier impression to the participants than that of the NPS (*M* = 2.6, *SE* = 0.07). As a result of Wilcoxon’s signed-rank test, significant differences were found (*p* < 0.001, effect size (*r*) = 0.49). In addition, the “Familiarity” rating was higher for PPS (*M* = 4.6, *SE* = 0.1) than that of NPS (*M* = 3.5, *SE* = 0.1) on the scale for driving support acceptance in the section “Acceptability Evaluation of Driving Support.” There was also a significant difference in this item (*p* < 0.001, *r* = 0.53). These results suggested that PPS was rated higher than NPS in the assessment items related to closeness. Therefore, the relationship between *D* expressed by the DSA (i.e., the expected effect of PPS/NPS in the experimental condition) and the evaluation of *D* by the experimental participants (mean value) is consistent with Figure 3.

### Result 1: Acceptability as the Driving Support

Herein, in order to analyze the relationship between utterance strategy and acceptability, the data collected in the section “Experiment” were classified into the following four groups: all participants (i.e., *All participants*, *n* = 197), the participants who appreciated PPS (i.e., *PPS group*, *n* = 134, male: 64, female: 70, average age = 38.9 years, *SD* = 9.4), the participants who appreciated NPS (i.e., *NPS group*, *n* = 32, male: 19, female: 13, average age = 35.6 years, *SD* = 8.1), and the participants who evaluated PPS and NPS equally (i.e., *Even group*, *n* = 31, male: 20, female: 11, average age = 37.7 years, *SD* = 11.6). The classification of the participant groups is based on the comparison of the results of the total score (minimum score is 9 and maximum score is 63) between PPS and NPS of each of the participant in all the nine items (*Cronbach’s alpha* = 0.94). The items used in the manipulation check (“Familiarity”) were also included in this analysis. We compared the total PPS scores of nine items (*Spps of Pi*) and total NPS scores (*Snps of Pi*) as assessed by a certain participant (*Pi, i*: 1–197) and grouped them according to the following procedure.

*If (Spps of Pi* > *Snps of Pi)*

Pi is in the PPS group

*else If (Spps of Pi* < *Snps of Pi)*

Pi is in the NPS group

else

Pi is in the Even group

We adopted this procedure to classify all the participants (*n* = 197) in order to ensure that all of them were included in the analysis. The ratio of the number of participants in each group was approximately 4:1:1. To examine the validity of these groups, an ordinal logistic regression analysis was conducted with the group to which each participant is assigned (PPS group, NPS group, and Even group) as the objective variable. The explanatory variables in this analysis were the ratings value for each of the nine items by participants in each experimental condition. As a result of the analysis, a significant model was obtained (*p* < 0.001). The coefficient of determination (Nagelkerke) of the model was 0.51, and the prediction accuracy of the objective variable was 72.08% (chance level is 33.3%). Based on these results, we believe that the group classification of the participants in this experiment is generally appropriate.

**FIGURE 6:**
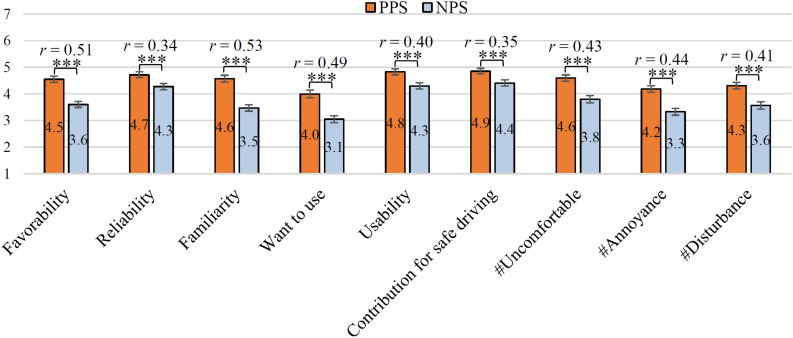
Acceptance of driving support by *All participants* (*n* = 197). ^∗∗∗^*p* < 0.001. The error bar shows the standard error.

**FIGURE 7:**
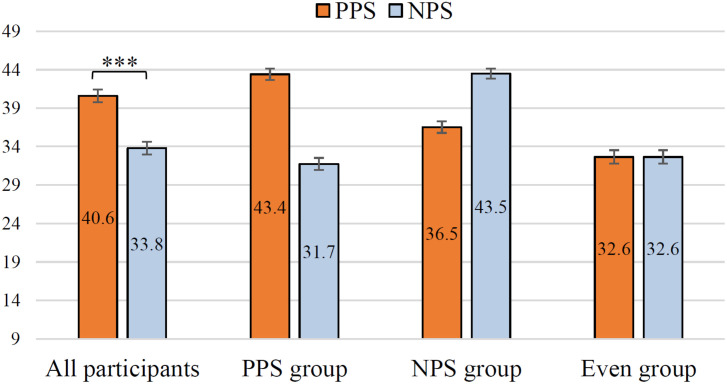
Acceptance of driving support by participant group. *All participants* (*n* = 197), *PPS group* (*n* = 134), *NPS group* (*n* = 32), and *Even group* (*n* = 31). ^∗∗∗^*p* < 0.001. The error bar shows the standard error.

Figure 6 shows the evaluation of the results of all items by *All participants*. In the figure, the higher the value on the vertical axis, the higher the acceptability of the utterance strategy. The Wilcoxon signed-rank test was performed to investigate whether there is a statistical difference in each item. As a result of the test, it was found that the evaluation of PPS was significantly high in all 9 items (i.e., *ps* < 0.001). It can be seen that *All participants* highly appreciated PPS. These *p*-values are corrected by the Bonferroni multiple-comparison correction method. In addition, *r* of each item is shown in Figure 6. Medium to large effect sizes (0.34 ≤ *r* ≤ 0.53) were obtained for all items. This suggests that the PPS is more acceptable than the NPS to many users. Figure 7 shows the evaluation results for each group. The vertical axis of this graph is the mean value of the total score of PPS and NPS for all 9 items. The mean of PPS is 6.8 points higher than the NPS in the *All participants*. The statistical analysis of PPS and NPS scores revealed a significant difference based on the Wilcoxon signed-rank test in *All participants* (i.e., *p* < 0.001, *r* = 0.57). Thus, a large effect size was obtained even when comparing PPS and NPS ratings in terms of the total score of the nine items.

### Result 2: Godspeed Questionnaire

**FIGURE 8:**
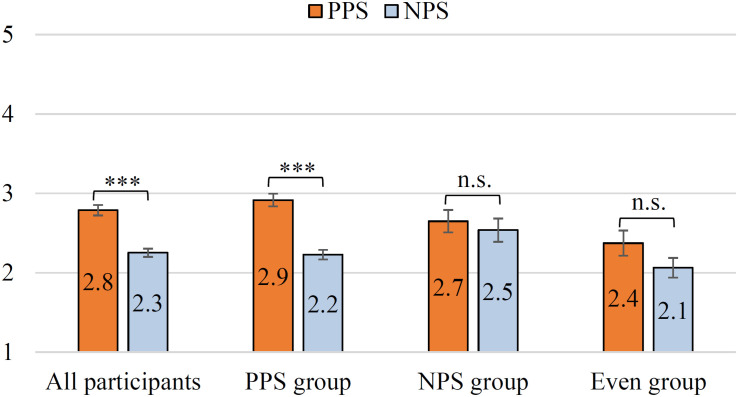
Impression of anthropomorphism. ^∗∗∗^*p* < 0.001. The error bar shows the standard error.

**FIGURE 9:**
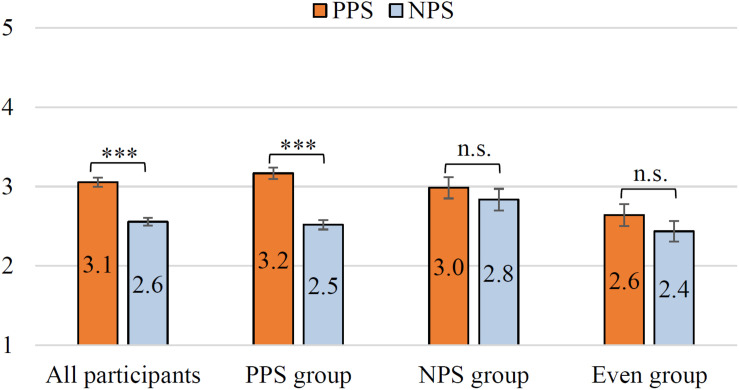
Impression of animacy. ^∗∗∗^*p* < 0.001. The error bar shows the standard error.

Based on the *All participants/PPS/NPS/Even group* classified in the section “Result 1: Acceptability as the Driving Support,” the results of the Godspeed scale are analyzed. In other words, we investigate the relationship between driving support acceptance and anthropomorphism/animacy/intelligence. When performing a significant difference test in this section, the *p*-value is corrected using the Bonferroni multiple-comparison correction method in consideration of the multiple comparisons for the four data groups. Figure 8 shows the evaluation results of anthropomorphism. The mean of the PPS is higher than that of the NPS in the *All participants*. The Wilcoxon signed-rank test showed significant differences in the *All participants* (i.e., *p* < 0.001, *r* = 0.50, stochastically significant after the Bonferroni multiple-comparison correction). This suggests that the PPS condition is more personified than the NPS condition for many users. Also, the PPS is higher than the NPS in the *PPS group*. Additionally, the test showed significant differences in *PPS group* (i.e., *p* < 0.001, *r* = 0.58, stochastically significant after the Bonferroni multiple-comparison correction). For the *NPS group* (*r* = 0.22) and *Even group* (*r* = 0.34), PPS was evaluated as better than that of NPS, but no significant difference was observed (i.e., *p* > 0.1). Figure 9 shows the evaluation results of animacy. The mean of the PPS is higher than that of the NPS in the *All participants*. In the *PPS group*, the mean of the PPS is also higher than that of the NPS. Similar to anthropomorphism, *All participants* (*r* = 0.47) and *PPS group* (*r* = 0.56) were significantly different for PPS vs. NPS (i.e., *p* < 0.001, stochastically significant after the Bonferroni multiple-comparison correction). In the *NPS group* (*r* = 0.22) and in the *Even group* (*r* = 0.34), the animacy of the PPS was evaluated as better than that of NPS, but the scores were not significantly different for PPS vs. NPS (i.e., *p* > 0.1). In addition, the *Spearman rank correlation coefficient* between driving support acceptance and the evaluation of anthropomorphism and animacy was about 0.1–0.4, and no strong correlation was observed.

**FIGURE 10:**
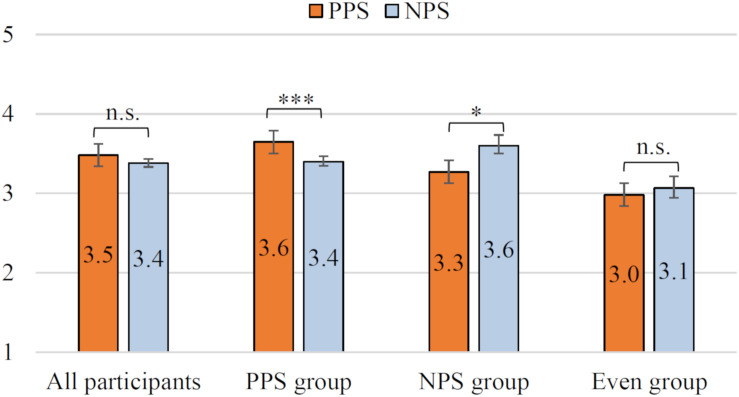
Impression of intelligence. ^∗∗∗^*p* < 0.001; ^∗^*p* < 0.05. The error bar shows the standard error.

Figure 10 shows the results of perceived intelligence. In the *All participants*, the mean of the PPS is higher than that of the NPS. Unlike anthropomorphism and animacy, there were no significant differences in the evaluation by *All participants* (i.e., *p* > 0.1, *r* = 0.10). On the other hand, there were significant differences between *PPS* and *NPS groups* (i.e., *PPS group p* < 0.001, *r* = 0.34; *NPS group p* < 0.05, *r* = 0.46, stochastically significant after the Bonferroni multiple-comparison correction). Specifically, in the *PPS group*, the mean of the PPS is higher than that of the NPS. Also, the mean of the NPS is higher than that of the PPS in the *NPS group*. The *Even group* was not significantly different (i.e., *p* > 0.1, *r* = 0.21). Therefore, for perceived intelligence, the PPS/NPS with high acceptability as driving assistance (in the section “Result 1: Acceptability as the Driving Support”) was highly evaluated in the *PPS/NPS group*. Furthermore, there was a strong correlation between the acceptance of driving assistance and the evaluation of intelligence (i.e., *Spearman rank correlation coefficient*, *p* < 0.001, NPS condition = 0.56, PPS condition = 0.69).

## Discussion

### Contribution to Agent Utterance Design

Compared to NPS, PPS was evaluated as the acceptable driving support utterance based on the evaluation by *All participants* (Figure 6). In addition, significant differences were observed in all 9 items used in the experiment, and large effect size was obtained from the medium (0.34 ≤ *r* ≤ 0.53). Therefore, as a whole, it is considered that PPS kept the face of the experimental participants compared with NPS. This result agrees with the experimental result of Miyamoto et al. (2019) targeting DSA. In previous studies (Salem et al., 2013; Torrey et al., 2013; Srinivasan and Takayama, 2016; Deshmukh et al., 2018; Lee et al., 2019), the agent’s utterance was designed based on the politeness strategies, but the difference in acceptability between PPS and NPS was not clear (i.e., no significant difference). However, this paper does not show a method for identifying the state of the user’s face. In a related study, a method for calculating *Wx* based on the user’s age, gender, and facial expression has been proposed (Miyamoto et al., 2020). In the future, by combining this method with the findings obtained in this paper, we can expect the development of a DSA that autonomously estimates *Wx* and selects an appropriate politeness strategy.

From the section “Result 1: Acceptability as the Driving Support,” the ratio of participants in the *PPS*/*NPS*/*Even group* was 4:1:1, respectively. In this study, this is called *Ratio of A*cceptability for *S*ocial distance *E*xpressed by *D*riving support agent (*Ratio of ASED*). The discovery of *Ratio of ASED* contributes mainly as an index for designing utterances in DSA. Specifically, the *PPS group* is a majority. However, it is difficult to decide on a single acceptable utterance strategy in developing a DSA. Therefore, we suggest that it is important to discuss an agent’s utterance to be implemented sufficiently from a viewpoint of verbal behavior. According to *Ratio of ASED*, in order to develop DSA that can be accepted by a wide range of users, it is important to design utterances considering that there are a certain number of *NPS group*, although they are minorities. The existence of the *NPS group* supports previous studies (Deshmukh et al., 2018; Lee et al., 2019). In previous studies, it has been shown that politeness strategies were more effective in improving acceptability than direct utterances. However, it was not shown that there was a user group in the acceptability of politeness strategies.

From the section “Result 2: Godspeed Questionnaire,” anthropomorphism and animacy were highly evaluated for PPS compared to NPS. This result is consistent with the result of Miyamoto et al. (2017), who suggested that PPS reduces the impression that a non-task-oriented conversational agent is a machine (i.e., increases the humanity of a non-task-oriented conversational agent). The effect of enhancing the agent’s humanity is thought to lead to a promotion of a phenomenon in which a user assigns an intention to an agent’s behavior and a user anthropomorphizes an agent (Dennett, 1989; Reeves and Nass, 1996; Miyamoto et al., 2017). Thus, increasing the anthropomorphism and animacy of an agent is a useful method to improve the interaction between the agent and the user. For example, if the DSA uses PPS to speak about the user’s driving, the user can be expected to attribute positive intentions to the DSA, which resultantly increases the affinity between the user and the agent. Also, since anthropomorphism and animacy are aspects of evaluation that have received much attention in the field of HRI (Bartneck et al., 2009), these results can be referred to for designing robots that interact with humans. However, there was no significant difference in the impression of intelligence among all participants. On the other hand, the PPS/NPS with high acceptability as driving assistance (in the section “Result 1: Acceptability as the Driving Support”) was highly evaluated in the *PPS/NPS group*. Furthermore, the strong correlation between the driving support acceptance and intelligence evaluation suggests that designing the behavior of an agent that allows the user to feel intelligence may increase the acceptance of driving support. Increasing agent intelligence also leads to improved reliability (Geven et al., 2006).

### Limitations

The knowledge gained through this study contributes to considering psychological acceptability when implementing utterances mainly to DSA. However, we could not give the participants a strong impression of trust and friendliness because the maximum value of the PPS that received a relatively high evaluation in each evaluation item on driving support was just under five points (section “Result 1: Acceptability as the Driving Support”). This is because it is considered that the utterance content is limited to the surrounding information and suggestions for driving. In order to solve this problem, it may be effective to implement various utterances for DSA, not limited to task-oriented utterances such as surrounding information and driving suggestions, specifically the agent to ask any questions to the user (e.g., “*How is your health?*”), or for the agent to utterances containing simile (e.g., “*You drive like a pro!*”). We believe that doing so may improve the acceptability of DSA. Questions and simile are known to lead to an expression of intelligence (Carnegie, 2006). As described in the section “Contribution to Agent Utterance Design,” giving the impression that an agent is intelligent to a user is effective in improving the acceptability of driving assistance. Furthermore, the viewing time of the videos was about 2 min each. Therefore, it is possible that the time of the experiment was not enough to give the participants a strong feeling of friendliness and reliability, e.g., the agent can receive six points or more (maximum score is seven) for the evaluation item in the section “Result 1: Acceptability as the Driving Support.” We compared our experimental results with those of Tanaka et al. (2018b), who evaluated the acceptability of driving assistance using the same scale as ours. As a result, our experimental results showed that the overall evaluation of agent is one point lower than Tanaka et al.’s (2018b). In the experiment conducted by Tanaka et al. (2018b), the number of times a participant listened to the agent’s utterance was three times higher than our experiment. Unlike our study, Tanaka et al. (2018b) also examined the effect of the number of contacts between the user and the agent, which is thought to affect the evaluation. Therefore, we suggest long-term experiments to verify the improvement in an agent’s acceptability by the mere exposure effect (Zajonc, 1968).

In this study, experiments were conducted only in Japanese; thus, it does not necessarily contribute directly to all languages and cultures. However, since politeness theory can be applied on other languages, it is possible to carry out the experiment for other languages. There are also politeness strategies and driving situations that have not been investigated in this study. Furthermore, the user segment of the *Ratio of ASED* is still unknown. By solving these problems, the usefulness of the *Ratio of ASED* can be further enhanced. On the other hand, in order to solve all these problems, we need to conduct experiments that take a huge number of variables into account.

Also, in this paper, the experimental stimulus was constructed only by a specific utterance set. Therefore, in this paper, the effects of PPS and NPS cannot be generalized. One of the solutions to this problem is to create a wide variety of utterances for each politeness strategy and conduct an experiment in which they are randomly presented to the participants. It may also be useful to set the use of honorifics as an independent variable. On the other hand, in DSA studies, there is little knowledge about the effect of the difference in wording on acceptability. Therefore, the discussion in section “Contribution to Agent Utterance Design” is considered to contribute as a finding for DSA research. The above experiment will be carried out as future work. In addition to this paper, there are other studies that have applied politeness theory to the design of dialogue agents (e.g., Srinivasan and Takayama, 2016; Lee et al., 2019). However, to apply the findings of these studies to other studies, it is necessary to clearly present how other researchers can create or utilize PPS and NPS utterances. For example, in the field of natural language processing, the development of a learning device that classifies the politeness of utterance sentences using a dataset labeled with politeness by an annotator (ordinary people) as teacher data for a large-scale dialogue log between people has been developed (Danescu-Niculescu-Mizil et al., 2013). It is thought that the development of such research will enable other researchers, who are unaware of politeness theory, to create utterance examples and dialogue systems that consider politeness.

We conducted an experiment to evaluate the psychological receptivity to a DSA’s utterance and obtained useful insights. As a next step, we would like to implement a DSA–user interaction experiment using a driving simulator (e.g., UC-win/road). This will facilitate an objective evaluation based on the user’s driving behavior, not just psychological acceptability. However, as described in the section “Introduction,” the purpose of this study is to investigate the psychological acceptance of DSA utterances, and this was accomplished through the experiments conducted in the section “Experiment.” The video-based experiments may be a shortcoming of this study. On the other hand, experiments using video have been suggested to be effective evaluation methods for psychological indicators in the field of Human–Robot Interaction (e.g., Syrdal et al., 2008; Rosenthal-von et al., 2013). In addition, a previous study (Cramer et al., 2008) adopted video-based experiments to evaluate the acceptability of DSA to elucidate the relationship between DSA type/driving context factors and DSA acceptability. Thus, we believe that the psychological acceptability of DSA can be adequately evaluated through video-based experiments. We thus believe that an experiment using a driving simulator is outside the scope of this study. We would like to conduct it as a separate study in the future.

## Conclusion

In this study, we discussed the influence of social distance expressed by DSA’s utterance on its psychological acceptability by the user. For the utterance design of the agent, we used PPS and NPS in the typical politeness strategy of expressing social distance in interpersonal relationships. The validity of the designed utterance was evaluated by the researchers specializing in politeness theory. Using the designed utterances, we created the videos supported by the agent. Participants watched the videos from the driver’s perspective. The experiment was conducted in which participants were recruited with crowdsourcing, and participants evaluated the psychological acceptability of the agent’s utterances by watching the videos. As a result, the overall evaluation by the participants was higher in PPS than in NPS. However, there were some participants who evaluated NPS significantly higher than PPS or evaluated both strategies to be equal. Specifically, the ratio of the participants who highly evaluated PPS, participants who highly evaluated NPS, and participants who evaluated PPS and NPS equally was 4:1:1 (i.e., *Ratio of ASED*). This result contributes mainly as an index for implementing utterances to DSA. In the future, we plan to conduct an objective evaluation based not only on psychological acceptability but also on driving behavior by conducting driving experiments using a driving simulator.

## Data Availability Statement

The datasets generated for this study are available on request to the corresponding author.

## Ethics Statement

Ethical review and approval was not required for the study on human participants in accordance with the local legislation and institutional requirements. Written informed consent for participation was not required for this study in accordance with the national legislation and the institutional requirements.

## Author Contributions

TM, DK, TT, HK, YY, and KF designed the research. TM, DK, YS, and MU performed the research. TM and DK wrote the manuscript. All authors contributed to the article and approved the submitted version.

## Conflict of Interest

The authors declare that the research was conducted in the absence of any commercial or financial relationships that could be construed as a potential conflict of interest.

## Footnotes

1 http://spring-fragrance.mints.ne.jp/aviutl/ (accessed 2019.12.10).

2 https://www.google.com/intl/ja_jp/forms/about/ (accessed 2019.12.10).

3 Society of Automotive Engineers of Japan, https://www.jsae.or.jp/01info/rules/kenkyu-rinri.html (accessed 2019.12.10).

4 Crowd works, https://crowdworks.jp/ (accessed 2019.12.10).
